# *Bacillus vallismortis* Bioextracts Combined with Cold Plasma Air for Post-Harvest Fungi Biocontrol in Tomato (*Lycopersicum solanum*)

**DOI:** 10.3390/biotech15020032

**Published:** 2026-05-09

**Authors:** Marisol Cruz Requena, Miguel A. Medina-Morales, Paola Cano Reséndez, Leonardo Sepúlveda Torre, Thelma K. Morales Martínez, Karina Reyes Acosta, Catalina Hernández Torres, Miriam Desiree Dávila Medina

**Affiliations:** 1 Agro-Environmental Biotechnology Group, School of Chemistry, Autonomous University of Coahuila, Saltillo 25280, Coahuila, Mexico; marisol.cruz@uadec.edu.mx; 2 School of Chemistry, Autonomous University of Coahuila, Saltillo 25280, Coahuila, Mexico; paola.cano@uadec.edu.mx (P.C.R.); ykreyes@uadec.edu.mx (K.R.A.); chernandez@uadec.edu.mx (C.H.T.); 3 Bioprocess and Microbial Biochemistry Group, School of Chemistry, Autonomous University of Coahuila, Saltillo 25280, Coahuila, Mexico; leonardo_sepulveda@uadec.edu.mx (L.S.T.); thelma_morales@uadec.edu.mx (T.K.M.M.)

**Keywords:** antagonism, shelf life, bioconservation, secondary metabolites, *Alternaria solani*

## Abstract

Post-harvest diseases caused by phytopathogenic microorganisms generate significant economic losses, particularly in tomato crops affected by *Alternaria solani*. This study evaluated the effectiveness of cold plasma combined with a bioextract of *Bacillus vallismortis* as a biological strategy to extend tomato shelf life. In vitro antagonism assays were performed by confronting *B. vallismortis* against *A. solani*. Additionally, shelf-life tests were conducted on tomatoes treated with *Bacillus* cells and *Bacillus* cell-free bioextract (BCFB), followed by inoculation with *A. solani* spores, assessing incidence, severity, weight loss, and microbiological parameters over time. Subsequently, tomatoes were treated with cold plasma in combination with BCFB and reevaluated. Results showed significant antagonistic activity, with *B. vallismortis* and BCFB inhibiting *A. solani* by 75% and 50%, respectively. In untreated tomatoes, BCFB reduced disease incidence to 66.66% and severity to scale 2, compared to 100% incidence and scale 5 severity in controls. The combined treatment with cold plasma and BCFB showed the highest effectiveness, completely inhibiting *A. solani* (0% incidence, scale 0 severity), with tomatoes remaining healthy after 25 days. These findings demonstrate that cold plasma combined with *B. vallismortis* represents an effective and sustainable alternative for controlling post-harvest phytopathogens and extending tomato shelf life.

## 1. Introduction

Foodborne diseases represent one of the most frequent causes of illness worldwide, resulting in millions of cases and numerous outbreaks each year. According to the World Health Organization, approximately 600 million people become ill annually due to the consumption of contaminated food, leading to more than 400,000 deaths globally [1]. These illnesses are primarily associated with microorganisms, including bacteria, viruses, fungi and parasites, which commonly reach the human body through contaminated food or water. Food products can become contaminated by microorganisms or harmful substances through accidental or fraudulent means during different stages of production, handling, or distribution, leading to infections such as food poisoning and toxinfections, which may also involve chemical contaminants present in food or water. Consequently, food and water serve as important vehicles for the dissemination of infectious diseases [2,3,4].

Preventive strategies must therefore focus on identifying and controlling potential hazards at each stage of the food production process to minimize risks to consumers. Food safety encompasses a set of actions aimed at guaranteeing the highest possible level of protection for food products, recognizing that access to safe and nutritious food in adequate quantities is essential for sustaining life and promoting good health. In addition to their impact on human health, microorganisms associated with food contamination also generate significant economic losses due to reduced crop yields and post-harvest deterioration [5,6].

The main producers of mycotoxins are fungi belonging to the genera *Aspergillus*, *Fusarium*, *Penicillium*, *Claviceps*, and *Alternaria*, whose occurrence and toxin production are more frequently reported in food and feed from developing countries due to climatic conditions, inadequate production practices, limited technologies, and poor storage conditions. However, mycotoxin contamination represents a global concern, as contaminated products can be distributed worldwide through international trade. In addition, most fruits and vegetables have a high moisture content, making them highly susceptible to microbial growth and post-harvest deterioration, a process that can be further accelerated by factors such as mechanical damage, rodent activity, inadequate storage temperature, and relative humidity. In this context, ensuring food safety requires the strict application of hygienic practices throughout the entire food chain, including production, processing, storage, transportation, and commercialization [7,8].

The food industry has devoted intense research efforts to counteract the emergence of outbreaks caused by foodborne pathogenic bacteria. Thermal processing is commonly employed as a preservation method, but intense thermal treatments can entail unwanted organoleptic and nutritional effects onto food [9]. There are chemical products that depend on specific types of microorganism to preserve food, as well as on the composition of the food and, above all, on the inhibition process. These preservatives demonstrate good efficiency, which is why they are considered adequate for maintaining food quality. However, they can cause problems for human health such as allergic reactions, intoxications and diseases. That is why biopreservation is sought since it is based on improving the microbiological safety of food, inhibiting unwanted microorganisms in the food with antimicrobial compounds [10,11]. Biopreservation strategies are those based on the use of natural substances derived from bacteria, fungi, plants or animals, with the aim of extending the shelf life of food products while guaranteeing their safety [9]. Biopreservation has emerged as a promising and sustainable alternative to conventional chemical preservatives, aiming to extend food shelf life using beneficial microorganisms and their antimicrobial metabolites [12,13]. In recent years, increasing concerns have been related to the potential adverse health effects of synthetic preservatives. In this context, biopreservation relies primarily on microorganisms such as lactic acid bacteria and *Bacillus* spp., which produce bioactive compounds including extracellular enzymes, nucleic acids, antibiotics, bacteriocins, organic acids, and lipopeptides capable of inhibiting the growth of pathogenic and spoilage microorganisms [14,15]. These compounds are generally recognized as safe (GRAS) and can be applied directly in food systems or through protective cultures and bioextracts. Moreover, biopreservation not only enhances microbiological safety but also contributes to maintaining sensory and nutritional quality, while reducing the reliance on synthetic additives. Therefore, the use of microbial-based preservation strategies represents an effective, eco-friendly, and innovative approach for controlling foodborne pathogens and reducing post-harvest losses in modern food systems [13].

Species of the genus *Bacillus*, such as *Bacillus subtilis* and *Bacillus cereus*, have been widely reported for their ability to produce a diverse range of lytic enzymes, including chitinases, proteases, lipases, and β-glucanases, which play a crucial role in the degradation of fungal cell walls and the inhibition of phytopathogenic fungi. In addition to enzymatic activity, *Bacillus* spp. is known to synthesize a variety of antimicrobial secondary metabolites, such as lipopeptides, including fengycin, iturin, and surfactin, which exhibit strong antifungal activity, particularly against filamentous fungi. These compounds act by disrupting fungal cell membranes and inhibiting spore germination, thereby contributing to the effective biological control of plant pathogens [15,16].

Cold plasma (CP) has recently emerged as a promising non-thermal technology for biological control in food systems, gaining increasing attention as an alternative to conventional decontamination methods. This technology offers several advantages for the food industry, including high efficiency in microbial inactivation, low processing temperatures, minimal impact on food quality, and applicability against a wide range of microorganisms, including spores and spoilage-related microbiota. Plasma is considered the fourth state of matter and is defined as a partially ionized gas composed of reactive species such as ions, electrons, neutral molecules, and radicals, which are responsible for its antimicrobial activity [17,18,19].

The use of cold plasma has great advantages, and one of them is its ecological benefit since it reduces the use of water, has no chemical residues, and the environmental air is used as a working gas. It has very little food penetration; only the surface is affected, so cold plasma helps retain nutrients and does not affect nutritional quality, nor does it show changes in the organoleptic characteristics of food [20]. The objective of this research was to evaluate the antagonistic activity of cold plasma in combination with *Bacillus* for the control of post-harvest fungi in tomatoes to extend their shelf life through an emerging biological and ecological technology.

## 2. Materials and Methods

### 2.1. Isolation of Bacillus spp.

A sampling of the rhizosphere of plants (prickly pear, walnut tree, apple tree, anthill soil, onion, cacti) was carried out in different areas of the state of Coahuila. This was performed at a depth of 10 to 20 cm, taking a sample of 30 g. Subsequently, 1 g of each sample was weighed and dissolved in 9 mL of sterile distilled water, subjected to a temperature of 50 °C for 10 min in a water bath, to eliminate saprophytic microorganisms such as fungi and bacteria; next, dilutions were made (1 × 10^−1^–1 × 10^−4^), and 1 mL of the last dilution was poured into a Petri dish with Potato Dextrose Agar medium enriched with 3% malt and yeast extract (PDA-ML3%) by simple diffusion. This was done in duplicate. The bacteria that showed typical *Bacillus* growth were isolated; from there, they were observed under a microscope and Gram staining was performed. Strains of *Bacillus* spp. were selected according to their macroscopic and microscopic morphological characteristics [21].

### 2.2. Preliminary Bioassays of Antagonism with Bacillus sp.

Selected strains identified as *Bacillus* spp. were evaluated by confrontation with the phytopathogenic fungus *Alternaria solani* in PDA-LM 3% medium. A smear from each strain of *Bacillus* spp. was set in the four cardinal points of each Petri dish; we placed a 4 mm diameter explant of the fungus *A. solani* at the center for its inhibition test. Plates were incubated at 28 °C for 5 to 10 d. Six repetitions were performed with a negative control without bacteria, and we quantified this by measuring the diameter of the diametral growth with a digital Vernier in the four cardinal points compared with the absolute control. A strain that showed antagonistic activity and permanence over time was selected.

### 2.3. DNA Extraction, Amplification by Polymerase Chain Reaction (PCR) and Molecular Identification

The bacterial DNA was isolated according to the protocol described below. Selected colonies were suspended in sterile distilled water in 1.5 mL microtubes for washing and centrifuged at 10,000 rpm for 10 min twice to discard the supernatant. Subsequently, the pellet was resuspended in 200 µL of TE (10 mM TRIS-HCl pH 8; 1 mM Na2EDTA), 200 µL of cell lysis solution (1 mM EDTA, SDS 2%, 200 mM NaCl, 20 mM Tris-HCl pH 8) and 10 µL of proteinase K. The samples were placed in the vortex mixer for 3 min and incubated for 30 min at 55 °C, then centrifugated at 10,000 rpm for 10 min. The supernatant was transferred to 1.5 mL microtube and mixed gently with half a volume of chloroform-isoamyl alcohol (24:1). The mixed samples were centrifugated at 10,000 rpm for 5 min; the aqueous phase was obtained and mixed with 200 µL of chloroform-isoamyl alcohol (24:1) and centrifugated under the same conditions. The supernatant was recovered and mixed gently by inverting for 1 min with 400 µL cold absolute isopropanol for DNA precipitation, and then the samples were incubated at −20 °C for 2 h and finally centrifugated at 10,000 rpm for 10 min. The supernatant was discarded. The DNA pellets were washed by adding 600 µL of 70% cold ethanol and centrifuged at 10,000 rpm for 10 min, followed by supernatant elimination and drying in air at room temperature; 50 µL of TE (10 mM TRIS-HCl pH 8; 1 mM Na2EDTA) was added to dried DNA followed by incubating at 65 °C for 60 min to accelerate hydration. Extracted DNA was stored at −20 °C for further use.

PCR amplification was carried out by the amplification of 16S rRNA gene with 27f (5′-AGA GTT TGA TCC TGG CTC AG-3′) and 1512r (5′-ACG GCT ACC TTG TTA CGA CTT-3′) primers with 50 µL PCR volume. PCR conditions were 95 °C for 10 min, 25 cycles of 93 °C for 1 min, 50 °C for 1 min, and 72 °C for 30 s, with a final extension cycle of 72 °C for 10 min. The final PCR product was viewed in agarose gel electrophoresis and visualized and photographed by a UV transilluminator. The PCR products were commercially sequenced by the Macrogen Laboratory (Rockville, MD, USA). The amplified 16S rRNA gene sequence was further used for the analysis of sequence similarity through the Basic Local Alignment Search Tool (BLAST) from the National Center for Biotechnology Information (NCBI). The nucleotide sequence was submitted on Bankit (NCBI) (BankIt) to get the specific accession number for the bacteria.

### 2.4. Antagonism Bioassays of the Bacillus and Cell-Free Bioextract (BCFB)

We inoculated the selected strain of *Bacillus* into 250 mL Erlenmeyer flasks with 60 mL of 3% potato-LM culture broth at a pH of 7.00, and these were cultured on a rotary shaker at 150 rpm for 7 d at room temperature. Upon obtaining growth of the bacteria and checking sporulation, we transferred samples to sterile conical tubes to be centrifuged at 3000 rpm for 15 min. The supernatants were filtered with 0.45 μm nitrocellulose membranes to obtain a (BCFB) cell-free filtrate [22]. Antagonism bioassays were performed by placing sterile filter paper in the four cardinal points of each Petri dish with 3% PDA-LM culture medium. Next, 5 μL of concentrated (100%) and 50% BCFB were added to each filter; a 5 mm diameter explant of *A. solani* was placed in the center for the inhibition test. Plates were incubated at 28 °C for 5 to 10 d. Six repetitions were performed with a negative control and were quantified by measuring the diameter of the growth with a digital Vernier in the two cardinal points compared with the absolute control [23].

### 2.5. Statistical Analysis

A completely randomized design with six repetitions was performed; different concentrations represented treatments and each Petri dish represented the experimental unit. Analysis of Variance (ANOVA) and Tukey’s mean comparison test *p* < 0.05 were performed to differentiate the antagonist activity of BCFB with respect to the growth obtained in the control.

### 2.6. Shelf Life of BCFB

Tomatoes obtained from a local business were disinfected with 3% sodium hypochlorite for three minutes, rinsed with sterile water, then dried. Tomatoes were inoculated by spraying with three treatments, concentrated BCFB, 75% and control (water); each treatment consisted of three repetitions of six tomatoes each. After the fruits were completely dried, a dilution of spores of *A. solani* (1 × 10^9^) was added by spraying. These were placed in plastic containers with sterile absorbent paper. Parameters such as weight, microbiological analysis, incidence and severity were recorded every 5 d (0, 5, 10, 15, 20, and 25).

#### 2.6.1. Weight

To examine the weight parameter, three tomatoes were randomly selected from each treatment and weighed during the days the experiment was conducted [24].

#### 2.6.2. Microbiological Analysis

Three tomatoes per treatment/repetition were ground and 1 g of the mixture was added to 9 mL of sterile peptone water (0.01%); then, we performed serial dilutions 10^−2^ to 10^−5^. An amount of 1 mL of the dilutions was added to Petri dishes; next, culture medium (poisoned medium), PDA for fungal isolation and nutritive agar for bacteria were and incubated at 28 ± 5 °C for 5 d for subsequent isolation and partial identification. The fungi that were present were isolated and identified by means of taxonomic keys of Barnett and Hunter (1998); bacteria were identified by Gram staining in all treatments, including the control [24].

#### 2.6.3. Incidence and Severity

This was analyzed on a scale of 1–5 (Table 1), measuring the damage caused by *A. solani* or by any other pathogenic microorganisms present; in the case of incidence, we counted the number of damaged tomatoes, and a percentage of incidence was estimated (Equation (1)):

(1) $$Percentage\;of\;Incidence\;(\%)=\frac{Number\;of\;diseased\;fruits}{Total\;fruits}\times 100$$

### 2.7. Shelf Life of Radiofrequency Cold Plasma with BCFB

Cold plasma treatment was performed using a dielectric barrier discharge (DBD) system operating at atmospheric pressure. The system consisted of an input voltage of 260 V (50 Hz) connected to a high-voltage transformer delivering up to 60 kV. Tomatoes were placed inside low-density polyethylene (LDPE) packages (1–2 mm thickness) filled with atmospheric air, which acted as both a sample holder and dielectric barrier between electrodes. The distance between electrodes was fixed at 47 mm.

The LDPE packages were sterilized prior to use by immersion in 70% ethanol followed by UV exposure for 20 min under a laminar flow hood. Samples were exposed to cold plasma for 5 min. Immediately after plasma treatment, tomatoes were sprayed with concentrated cell-free bioextract (BCFB) derived from *Bacillus* sp. under aseptic conditions. After drying, fruits were inoculated by spraying with a spore suspension of *Alternaria* sp. adjusted to 10^9^ spores/mL [25].

Treated tomatoes were placed in plastic containers with moist sterile absorbent paper to maintain humidity and stored at 27 ± 2 °C under controlled conditions for 25 d.

Shelf-life evaluation was performed every 5 d (0, 5, 10, 15, 20, and 25 days), assessing disease incidence and severity according to a previously established scale. All treatments were performed in triplicate (*n* = 3), with six fruits per replicate.

Tomatoes treated with cold plasma were sprayed with concentrated BCFB under aseptic conditions, and when completely dry, they were inoculated with a solution of *A. solani* spores (1 × 10^9^) by spraying. They were placed directly in a plastic container with sterile damp absorbent paper. A shelf-life test was performed only measuring incidence and severity. They were left at room temperature with humidity during the 25 d that the experiment lasted and evaluated every 5 d; at the end of this span, we checked for post-harvest diseases caused by fungi and/or presence of any other alteration.

### 2.8. Qualitative Determination of Chitinases from Bacillus vallismortis

Tests were carried out in a minimal medium (0.003% NaCl, 0.03% MgSO_4_ and 0.015% K_2_HPO_4_). And the presence of chitinase was determined qualitatively in a Petri box.

Determination of chitinase activity: Colloidal chitin medium was used. It was sterilized, then it was emptied into plates; it was expected to solidify and then it was sown in the middle of plate *Bacillus*. They were incubated at 28 °C for 10 d [26].

## 3. Results and Discussion

### 3.1. Isolation and Identification of Bacillus

**Figure 1:**
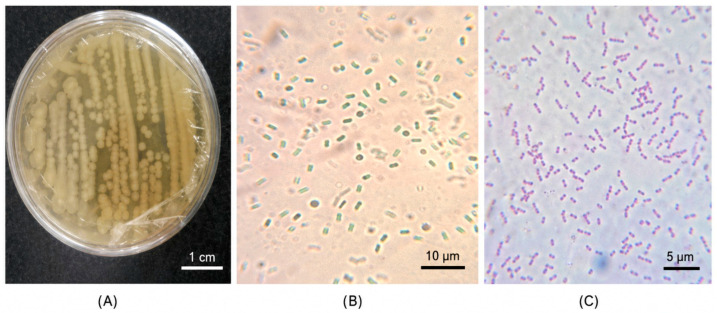
Macroscopic and microscopic morphology of *Bacillus*. (**A**) Colonies of *Bacillus* Macroscopic Morphology. (**B**) Spores of *Bacillus* Microscopic Morphology. (**C**) Stain, Gram-positive.

Seven characteristic strains of *Bacillus* spp. were obtained, isolated from prickly pear cactus, walnut, apple, onion, anthill soil, and cacti. The isolated colonies showed a rough, opaque appearance, irregular border, dry-looking surface and yellow-white coloration. Microscopically, they were Gram-positive bacilli, with the capacity to form endospores (0.6 to 0.9 by 1.2 to 1.5 microns) that do not deform the *Bacillus* (Figure 1) [27].

In antibiosis bioassays, high inhibition percentages against *Alternaria solani* were obtained in all strains isolated from soil, ranging from approximately 50% to 80%. The selected strain was isolated from the prickly pear cactus rhizosphere. It exhibited the characteristic morphology of *Bacillus* and the highest percentages of antagonistic activity against *Alternaria solani* (73%), with sustained activity over time. It was subsequently identified molecularly.

As for the molecular identification, the results were subjected to BLAST analysis using the NCBI database to identify genus and species. Isolates had different nucleotide sequences for the DNAr 16S gene, indicating that the selected isolate is closely related to *Bacillus vallismortis* with 99% similarity (Table 2).

### 3.2. Preliminary Antagonism Bioassays of Bacillus vallismortis Against A. solani

**Figure 2:**
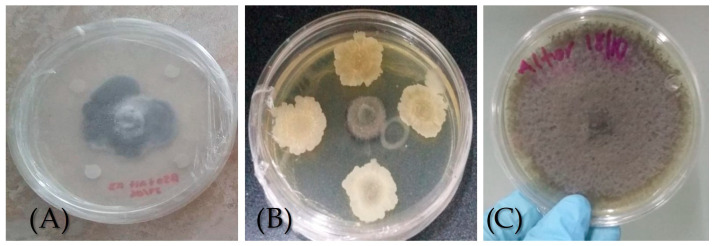
Antagonism bioessays of *B. vallismortis* against *A. solani*. (**A**) Antagonistic effect of BCFB. (**B**) Antagonistic effect of *B. vallismortis* (**C**) *A. solani* control.

*Bacillus vallismortis* inhibited the growth of the phytopathogenic fungus *A. solani* in PDA-LM 3% medium by up to 70% (Figure 2, Table 3). This agrees with reports of Castillo-Reyes et al. [21] who obtained similar results (up to 67% of inhibition) against phytopathogenic fungi, such as *Fusarium* and *Rhizoctonia.* Several authors confirm the antagonistic effectiveness of *Bacillus* sp. against phytopathogenic fungi such as *Colletotrichum*, *Rhizoctonia*, *Fusarium*, *Phytiumi*, etc. [28]. Touré et al. [29] stated that *Bacillus subtilis* is an effective biocontrol agent that produces fengycines which protect apples against the pathogen *Botrytis cinerea*. Its antagonist activity has also been proven in further post-harvest diseases of apples and pears [30]. These metabolites act by disrupting fungal cell membranes, increasing permeability, and inhibiting spore germination, ultimately leading to cell death. Furthermore, the higher efficacy observed in the concentrated treatment suggests a dose-dependent effect, where increased concentrations of antimicrobial compounds enhance fungal inhibition [12,15].

### 3.3. Bioassays of Antagonism of the Bacillus Cell-Free Bioextract (BCFB) Against A. solani

Both the concentrated BCFB concentrate and BCFB at 75% presented 52.2% and 48.7% of percent inhibition, respectively (Table 3). This difference was not significant. However, compared to the control there was a statistically significant difference since, in the control, 0% inhibited was observed (Figure 2). The results obtained are similar to those reported by Arroyave et al. [31] who performed in vitro bioassays with *B. subtilis* and *B. subtillis* cell-free supernatant, showing that both inhibited phytopathogens. They attributed this effect to the lipopeptides iturin A and fengycin C which reduce fungal development and are present in the supernatant free of *B. subtillis* cells.

### 3.4. Shelf Life of BCB—Treated Tomatoes

#### 3.4.1. Weight

During the initial days of storage at room temperature, no significant differences in weight loss were observed between treatments and the control. However, by day 15, a marked decrease in weight was recorded in control due to advanced fruit degradation, whereas tomatoes treated with *B. vallismortis* maintained a more stable weight throughout the experimental period. This effect may be associated with the ability of *Bacillus*-derived metabolites to delay ripening and reduce physiological deterioration processes. Weight loss in fresh produce is primarily related to water loss through transpiration and respiration, which are closely linked to the metabolic activity and structural integrity of the fruit. The reduced weight loss observed in treated tomatoes suggests that microbial metabolites may contribute to preserving cell wall integrity and reducing respiration rates, thereby slowing down senescence [32,33].

#### 3.4.2. Microbiological Analysis

**Figure 3:**
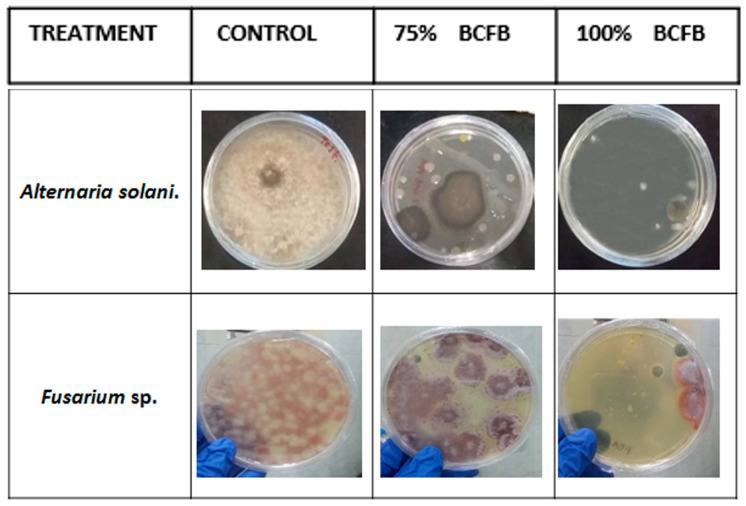
Microbiological analysis of the shelf life of tomatoes treated with BCFB in PDA for fungi.

The results revealed clear differences among the evaluated treatments (control, concentrated, and 75%) (Figure 3). Compared to the control, the concentrated treatment showed the highest inhibitory effect against *Alternaria solani* as well as against another fungus identified as *Fusarium* sp., indicating a strong antifungal activity of the cell-free bioextract (BCFB). The bacterial colonies observed are not *Bacillus* growth, but rather saprophytic microorganisms naturally present on tomatoes. This was verified based on both macroscopic and microscopic morphological characteristics, which differ from those of the *Bacillus* strain used in this study. *Bacillus* spp. have been extensively studied as biological control agents of plant pathogens, as several strains produce a wide range of antimicrobial metabolites. Among these, lipopeptides (LPs) represent a rich source of diverse bioactive compounds with a wide range of industrial applications [34]. Lipopeptides such as fengycin, iturin, and surfactin, which are known for their broad-spectrum antifungal properties, have been studied as natural bio-preservatives due to their high effectiveness, low toxicity, and environmentally friendly characteristics. Fengycin, with strong antifungal activity, has been evaluated as a potential natural preservative to curb apple ring rot disease by inhibiting *Botryosphaeria dothidea* [35].

#### 3.4.3. Incidence and Severity

**Figure 4:**
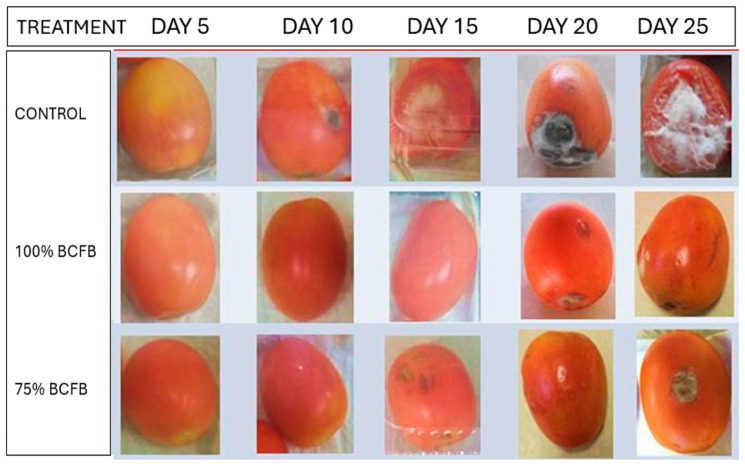
Severity scale of tomato shelf life after 25 days of BCFB treatment.

The evaluation of disease severity during the shelf-life assay revealed statistically significant differences between the control and all treatments. At the end of the experimental period, the control exhibited the highest level of disease development (scale 5), whereas tomatoes treated with *Bacillus vallismortis* showed a reduction in severity, reaching scale 2 in the 50% treatment and scale 1 in the concentrated treatment (Figure 4) (Table 4).

Similarly, disease incidence showed a clear trend over time, with the control reaching 100% infection from day 5 until the end of the trial. In contrast, the 75% and concentrated treatments reduced incidence to 83.32% and 66.66%, respectively, confirming a significant decrease in infection levels (Figure 4) (Table 5).

The lower incidence and severity observed in treated fruits suggests that secondary metabolites of *B. vallismortis* may act at early stages of pathogen establishment, potentially inhibiting spore germination and initial infection processes. Previous studies have reported that biological control agents can effectively reduce both incidence and severity of post-harvest diseases by limiting pathogen establishment and slowing disease progression under storage conditions [36].

### 3.5. Shelf-Life Test of Tomato Treated with Cold Plasma and BCFB

#### Incidence and Severity

**Figure 5:**
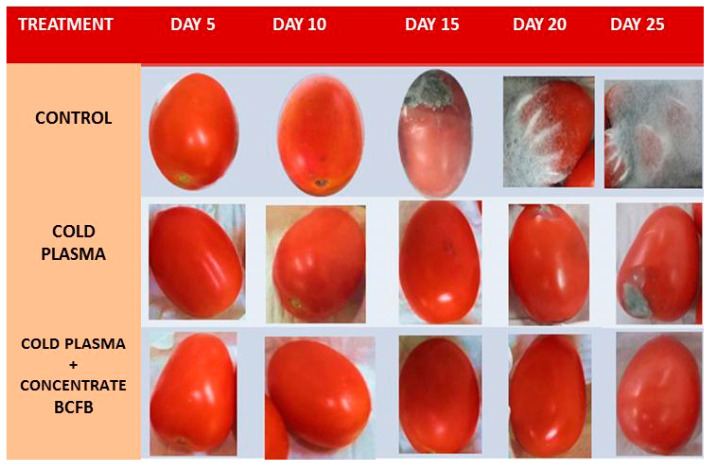
Severity of shelf life of tomatoes treated with cold plasma with BCFB after 25 days of treatment.

At 25 d of the trial, the incidence in the control was 100%, in the cold plasma treatment it was 66.66%, while in the cold plasma treatment + BCFB it was 0% until the last day of testing; the modified tomatoes were healthy (Table 6). There was a statistically significant difference compared to the control. Regarding the severity, there was also a significant difference compared to the control, which was completely degraded by fungi and bacteria, showing scale 5 severity. In contrast, tomatoes treated with cold plasma with BCFB were completely healthy at day 25 of the trial, i.e., a 0 on the severity scale (Table 7) (Figure 5).

This enhanced efficacy may be attributed to the surface modifications induced by cold plasma on tomato tissues. Cold plasma generates reactive species capable of altering the microstructure of plant surfaces, leading to partial erosion, disruption of epidermal cells, and increased surface roughness and hydrophilicity. These structural changes can improve the interaction between the fruit surface and bioactive compounds. In a previous study, fruits and vegetables treated with plasma were inoculated with pathogenic microorganisms; the authors observed significant reductions in severity of infection according to the duration of treatment [36,37,38].

Although cell-free bacterial extracts from *Bacillus* spp. have shown promising antifungal activity, their safety for direct application on fresh produce intended for human consumption remains an important consideration. Several studies have reported that certain *Bacillus* strains, particularly those used as probiotics, are generally regarded as safe due to their non-pathogenic nature and absence of toxin production, as well as their lack of adverse effects in acute and chronic toxicity studies [39].

In addition, lipopeptides produced by *Bacillus* spp. are often described as biodegradable and low-toxicity compounds with potential applications in the food and pharmaceutical industries; however, available toxicological data remain limited and may vary depending on the strain and concentration used [34]. Recent studies have also reported low or negligible toxicity of these metabolites in biological systems, supporting their potential as safe biocontrol agents [16].

### 3.6. Qualitative Determination of Chitinases from Bacillus vallismortis

**Figure 6:**
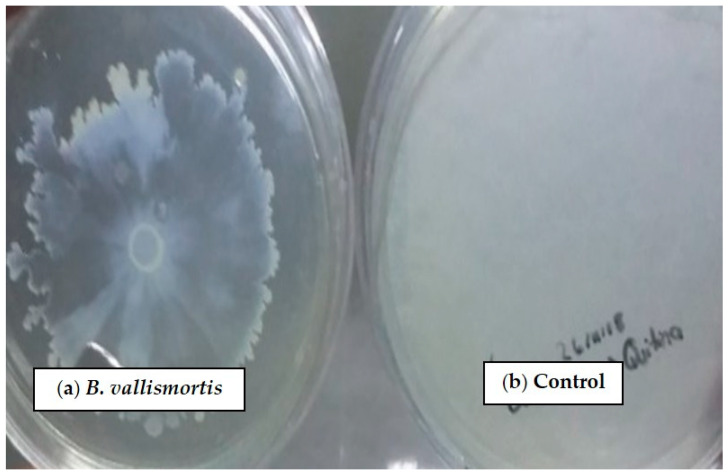
Qualitative determination of chitinases. (**a**) Growth of *Bacillus* with chitin hydrolyzing activity. (**b**) Compared with the control without hydrolysis.

We determined that *B. vallismortis* produces chitinases, since a clear halo was observed around the growth of the strains after the incubation period, hydrolyzing the chitin of the medium (Figure 6). Mayer and Kronstad [40], through genetic and phenotypic analyses, demonstrate that chitinase activity is a factor contributing to the destabilization of the fungal cell wall by *Bacillus*.

## 4. Conclusions

The soil of the rhizosphere is an important source of the genus *Bacillus* which has high potential as a biological control of phytopathogens. The *Bacillus vallismortis* cell-free bioextract turned out to be efficient in lengthening the shelf life of the tomatoes, reducing the levels of incidence and severity of diseases caused by phytopathogens such as *Alternaria solani*. The antagonistic activity of *Bacillus vallismortis* can be attributed to the different secondary metabolites that it produces, such as chitinases. The emerging technology of cold plasma in combination with bioextracts of *Bacillus vallismortis* potentiated the inhibitory effect on post-harvest phytopathogens in the shelf-life test with tomatoes, as these remained healthy after 25 d of testing. This was in stark contrast with the control which was totally decomposed. These findings highlight the potential of combining biological and physical strategies to improve post-harvest disease management. The proposed approach represents a sustainable and eco-friendly alternative to conventional chemical treatments, capable of extending shelf life while maintaining the nutritional and organoleptic quality of tomatoes. Future research should focus on elucidating the interaction mechanisms between cold plasma and microbial metabolites, particularly regarding surface modification and enhanced metabolite adhesion, as well as scaling up this technology for industrial post-harvest applications.

## Figures and Tables

**Table 1:** Severity scale for *A. solani* on tomato fruit.

Scale	Damage %
1	0–20
2	21–40
3	41–60
4	61–80
5	81–100

**Table 2:** Sequence submission of isolated bacterial identified through 16S rRNA amplification.

Strain ID on Bankit	Assigned Accession Number	Bacterial Name	Query Coverage (%)	Percentage Identity	E-Value
BM32	PZ323972	*Bacillus vallismortis*	100%	99%	0.00 × 10^0^

**Table 3:** Percentage of inhibition of *Bacillus vallismortis* and its cell-free filtrate BCFB against *Alternaria solani*.

Treatment	Percentage of Inhibition % *
	*Alternaria solani*
*B. vallismortis*	72.71 ^A^ ± 1.71
BCFB 100%	52.23 ^B^ ± 0.77
BCFB 75%	48.65 ^B^ ± 1.02
Control	0 ^C^ ± 0.00

* Values are expressed as mean ± standard deviation (*n* = 6). Different letters indicate significant differences according to Tukey’s test (*p* ≤ 0.05).

**Table 4:** Severity scale of *A. solani* infection on shelf life of tomato treated with BCFB.

Treatment	Severity Scale (% Damage) *				
	Day 5 *	Day 10 *	Day 15 *	Day 20 *	Day 25 *
Control	2 ^A^ ± 0.63	2.5 ^A^ ± 0.83	3.2 ^A^ ± 0.51	4 ^A^ ± 0.63	5 ^A^ ± 0
BCFB Concentrated	0 ^B^ ± 0	2 ^A^ ± 0	2 ^B^ ± 0	2 ^B^ ± 0	2 ^C^ ± 0
BCFB 75%	2 ^A^ ± 0	2.1 ^A^ ± 0.40	2.3 ^B^ ± 0.51	2.6 ^B^ ± 0.51	3 ^B^ ± 0.63

* Values are expressed as mean ± standard deviation (*n* = 18). Different letters indicate significant differences according to Tukey’s test (*p* ≤ 0.05).

**Table 5:** Incidence of *A. solani* infection on shelf life of tomatoes treated with BCFB.

Treatment	Day 5 *	Day 10 *	Day 15 *	Day 20 *	Day 25 *
Control	33.33% ^A^	66.66% ^A^	83.33% ^A^	100% ^A^	100% ^A^
BCFB Concentrated	0% ^C^	33.33% ^B^	33.33% ^C^	50% ^B^	66.66% ^B^
BCFB 75%	16.66% ^B^	50% ^A^	66.66% ^B^	66.66% ^B^	83.32% ^A^

* Different letters indicate significant differences according to Tukey’s test (*p* ≤ 0.05).

**Table 6:** Incidence of *A. solani* on the shelf life of tomatoes treated with cold plasma and BCFB.

Treatment	Day 5 *	Day 10 *	Day 15 *	Day 20 *	Day 25 *
Control	33.33% ^A^	66.66% ^A^	83.33% ^A^	100% ^A^	100% ^A^
Cold plasma	0% ^B^	16.66% ^B^	50% ^B^	66.66% ^B^	66.66% ^B^
Cold Plasma + BCFB	0% ^B^	0% ^C^	0% ^C^	0% ^C^	0% ^C^

* Different letters indicate significant differences according to Tukey’s test (*p* ≤ 0.05).

**Table 7:** Severity scale of *A. solani* in the shelf-life test of tomatoes treated with cold plasma and BCFB.

Treatment	Severity scale (% Damage) *				
	Day 5 *	Day 10 *	Day 15 *	Day 20 *	Day 25 *
Control	2 ^A^ ± 0	2.7 ^A^ ± 0.81	3.1 ^A^ ± 0.52	5 ^A^ ± 0	5 ^A^ ± 0
Cold Plasma	0 ^B^ ± 0	2 ^A^ ± 0	2.1 ^B^ ± 0.40	2.2 ^B^ ± 0.51	2.4 ^B^ ± 0.83
Cold Plasma + BCFB	0 ^B^ ± 0	0 ^B^ ± 0	0 ^C^ ± 0	0 ^C^ ± 0	0 ^C^ ± 0

* Values are expressed as mean ± standard deviation (*n* = 18). Different letters indicate significant differences according to Tukey’s test (*p* ≤ 0.05).

## Data Availability

The original contributions presented in this study are included in the article. Further inquiries can be directed to the corresponding authors.
